# Genomic predictions and genome-wide association studies based on RAD-seq of quality-related metabolites for the genomics-assisted breeding of tea plants

**DOI:** 10.1038/s41598-020-74623-7

**Published:** 2020-10-15

**Authors:** Hiroto Yamashita, Tomoki Uchida, Yasuno Tanaka, Hideyuki Katai, Atsushi J. Nagano, Akio Morita, Takashi Ikka

**Affiliations:** 1 Faculty of Agriculture, Shizuoka University, 836 Ohya, Suruga-ku, Shizuoka, 422-8529 Japan; 2 United Graduate School of Agricultural Science, Gifu University, 1-1 Yanagito, Gifu, 501-1193 Japan; 3 Shizuoka Prefectural Research Institute of Agriculture and Forestry, Tea Research Center, 1706-11 Kurasawa, Kikugawa, Shizuoka 439-0002 Japan; 4 Faculty of Agriculture, Ryukoku University, 1-5 Yokotani, Seta Oe-cho, Otsu, Shiga 520-2194 Japan; 5 Institute for Tea Science, Shizuoka University, 836 Ohya, Shizuoka, 422-8529 Japan; 6 Present Address: Shizuoka Prefecture Chubu Agriculture and Forestry Office, 2-20 Ariake-cho, Suruga-ku, Shizuoka, 422-8031 Japan

**Keywords:** Biotechnology, Computational biology and bioinformatics, Genetics, Plant sciences

## Abstract

Effectively using genomic information greatly accelerates conventional breeding and applying it to long-lived crops promotes the conversion to genomic breeding. Because tea plants are bred using conventional methods, we evaluated the potential of genomic predictions (GPs) and genome-wide association studies (GWASs) for the genetic breeding of tea quality-related metabolites using genome-wide single nucleotide polymorphisms (SNPs) detected from restriction site-associated DNA sequencing of 150 tea accessions. The present GP, based on genome-wide SNPs, and six models produced moderate prediction accuracy values (*r*) for the levels of most catechins, represented by ( −)-epigallocatechin gallate (*r* = 0.32–0.41) and caffeine (*r* = 0.44–0.51), but low *r* values for free amino acids and chlorophylls. Integrated analysis of GWAS and GP detected potential candidate genes for each metabolite using 80–160 top-ranked SNPs that resulted in the maximum cumulative prediction value. Applying GPs and GWASs to tea accession traits will contribute to genomics-assisted tea breeding.

## Introduction

Tea plants (*Camellia sinensis* L.) are mainly cultivated in Asia to produce green, oolong, and black tea, which are popular beverages throughout the world. Approximately two billion cups of tea are consumed daily worldwide^1^, and tea drinking reportedly has numerous health benefits^2^. In general, tea quality is defined by the balance of various functional components, such as theanine, catechins, and caffeine, which are unique to tea. Theanine, an amino acid, contributes to the major *umami* taste of green tea^3,4^, and it has various health benefits, such as promoting relaxation^5^, improving concentration and learning ability^6^, and reducing blood pressure^7^. Tea catechins are major polyphenols and have been studied for their anticarcinogenic effects^8^, antimutagenic effects^9^, antibacterial activities^10^, free radical scavenging activities^11^, and anticaries actions^12^. In particular, ( −)-epigallocatechin gallate (EGCG), the major component of tea catechins, has strong antiallergic effects^12,13^. Caffeine (1,3,7-trimethylxanthine) is a kind of purine alkaloid that accumulates to high levels in tea leaves and coffee. Caffeine consumption may be associated with a reduced risk for type 2 diabetes^14^, but excessive intake of caffeine may cause inflammation of the digestive organs, insomnia, and arrhythmia^15^. There are also reports on the risks of pregnant women ingesting caffeine^16^. These potentially harmful effects of caffeine negatively affect the consumption of tea and tea-related products. Thus, the unique metabolites in tea produce the most important agronomic traits targeted by modern and future tea breeding.

Conventional breeding based on phenotypic evaluations has many drawbacks, including the long generation time and large physical size of tea plants, and the inability to assess the marketable product prior to the tea seedling reaching physiological maturity. However, for tea plants and fruit trees^17^, traditional breeding methods are still commonly used. This must change to meet the demands of the expanding global market; therefore, tea breeding needs to be updated using new technology. Recent genomics-based approaches may be especially useful for increasing crop breeding efficiency^18^. The use of genomics-assisted breeding facilitates more selection cycles and greater genetic gains per unit of time. In particular, genomics-assisted breeding is effective for woody plants, such as tea and fruit trees, which have long life cycles. Additionally, the genotypic data obtained from seeds or seedlings in breeding populations can be used to predict the phenotypic performance of mature individuals without the need for extensive phenotypic evaluations in different years and environments.

Marker-assisted selection (MAS) and genomic prediction (GP) are the two main types of genomics-assisted breeding^18^. MAS uses molecular markers that map within specific genes or quantitative trait loci (QTLs) associated with target traits or phenotypes to select individuals that carry favourable alleles for the traits of interest. MAS is efficient for traits that are controlled by low numbers of QTLs that have major effects on trait expression, but it is not suitable for evaluating complex quantitative traits that are governed by large numbers of minor QTLs^19,20^. GP uses all the available marker data for a population as predictors of breeding value based on modelling, and it takes into account the effects of multiple genes that control a target trait; this overcomes the limitation of MAS^19^, although GP requires cost-effective high-throughput genotyping platforms.

Next-generation sequencing (NGS) technologies have drastically reduced the cost and time of sequencing and single nucleotide polymorphism (SNP) discovery, which led to the development of high-throughput genome-wide SNP genotyping. In particular, the emergence of restriction site-associated DNA sequencing (RAD-seq) and genotype-by-sequencing resulted from the implementation of SNPs suitable for GP in both model and non-model crops^17,18,21^. These high-throughput genome-wide SNP genotyping platforms have enabled the use of genome-wide association studies (GWASs) and GPs in many important crops^22–26^. GWASs enable detection of QTLs or genes that control phenotypic variations in a population of cultivars or germplasm accessions without preparing a bi-parental segregating population^27^.

Previously, we conducted SNP genotyping of 167 tea accessions and revealed the genetic structures of cultivars, landraces, and germplasm accessions for subsequent genomics-assisted breeding^28^. Very recently, the draft genomes of two major tea varieties, *C*. *sinensis* var. *assamica*^29^ and *C*. *sinensis* var. *sinensis*^30,31^, were sequenced using several NGS platforms. The genome size of tea plants was estimated to be 3.8–4.0 Gb^32,33^, and this size has been a major barrier to determining tea genomic information using NGS technologies. For *C*. *sinensis* var. *sinensis*, a high-quality chromosome-level reference genome was obtained using single-molecule real-time sequencing and chromatin conformation capture technologies^31^. This information enabled determination of the chromosomal positions of SNP markers and linking genes in the tea genome, and it was effectively used for GP and GWAS.

In the present study, we evaluated the potential of performing integrated analysis by GP and GWAS for genetic improvement of tea quality-related metabolites using genome-wide SNPs from RAD-seq data. Our analysis showed that GP effectively predicted the contents of several catechins and caffeine, but not free amino acids (FAAs). In addition, integrated analysis of GWAS and GP detected the potential candidate genes for each metabolite.

## Results

### Variations in 19 tea quality-related metabolites

**Figure 1:**
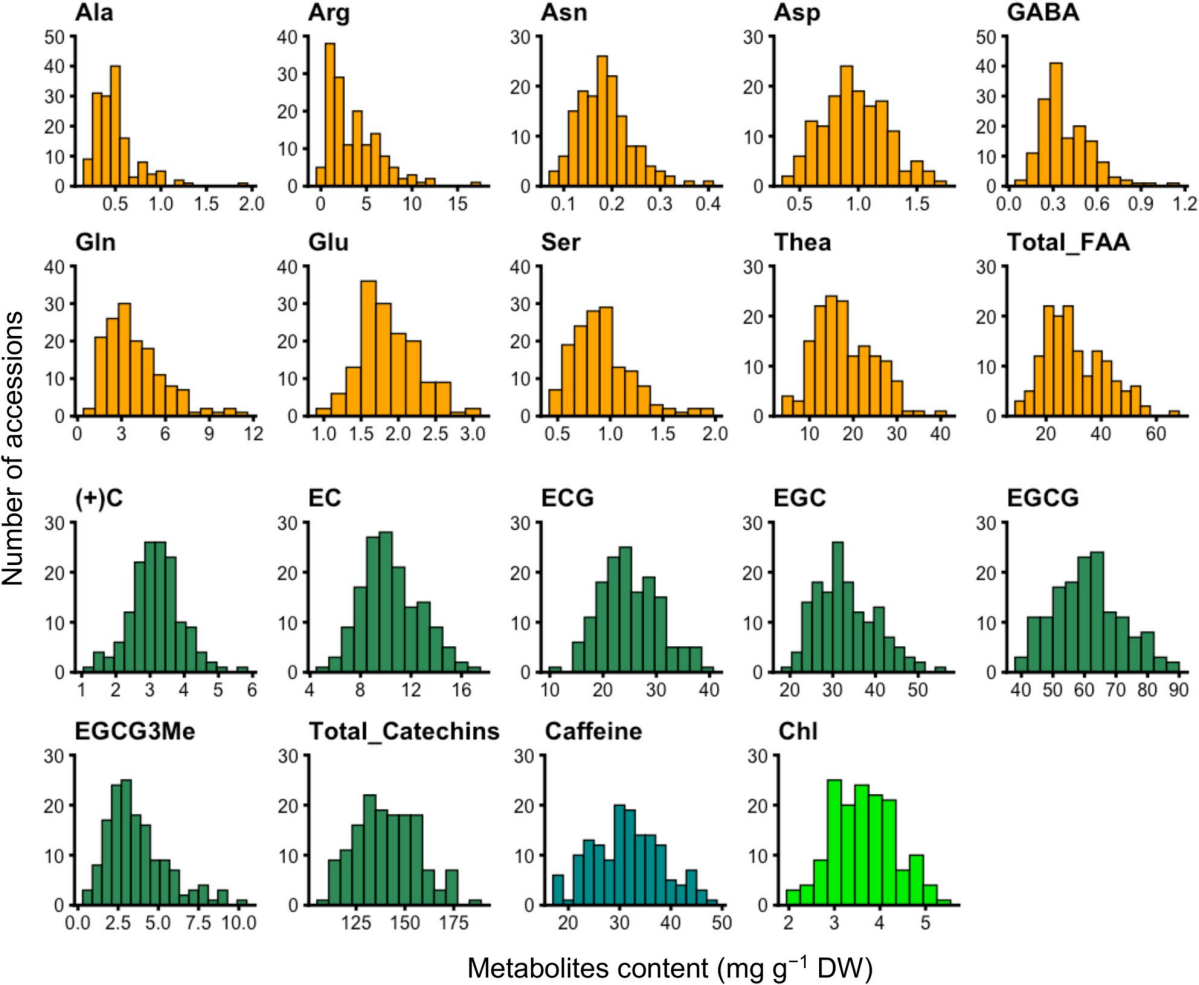
Phenotypic variations in the tea quality-related metabolites of 150 accessions.

To evaluate variations in the tea quality-related metabolites of tea accessions, we quantified the contents of 19 metabolites, including 10 FAAs, 7 catechins, caffeine, and chlorophyll (Chl), in the new shoots from the first crop season. We investigated the contents in 2018 and 2019 to evaluate the annual effect. Most variations were positively correlated between 2018 and 2019 (Supplementary Fig. S1). Therefore, the mean values obtained from 2018 and 2019 were used as values in the subsequent GP modelling and GWAS (Fig. 1).

### Genotyping using the latest chromosome-scale genome and linkage disequilibrium (LD)

Our previous study^28^ detected SNPs in these tea accessions using a scaffold-scale reference genome^30^, but the latest chromosome-scale reference genome was published by Xia et al. (2020)^31^. We re-detected SNPs using the chromosome-scale reference genome. After filtering [SNP call rate within a locus ≥ 0.7, minor allele frequency (MAF) ≥ 0.05], 9523 SNPs were detected for subsequent analyses and widely mapped across the whole genome (Supplementary Fig. S2).

**Figure 2:**
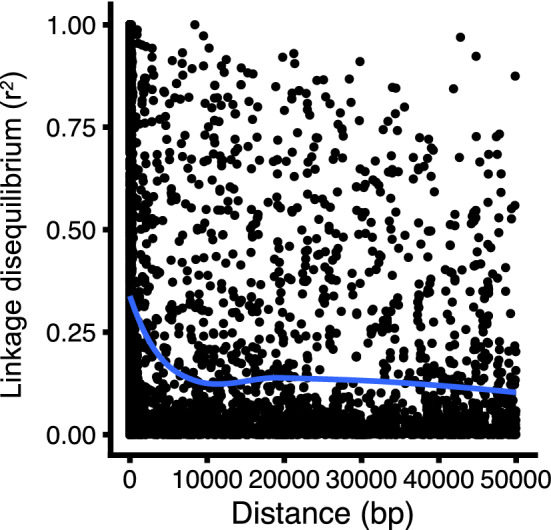
Estimates of linkage disequilibrium (LD) over genetic distance for all chromosomes of 150 tea accessions. The blue curve indicates the LD decay pattern that was estimated by fitting a trend line based on a nonlinear LOESS regression of *r*^*2*^ on physical distance.

The squared correlation coefficient (*r*^2^) values of the pairwise LD were plotted using a nonlinear regression curve against physical distance to estimate the LD decay pattern. This regression curve pattern showed that LD decayed to relatively low levels (*r*^2^ < 0.13) within 10 kb (Fig. 2). The mean LD between adjacent SNPs was *r*^*2*^ = 0.24.

### Accuracy of GP models for 19 tea quality-related metabolites

**Figure 3:**
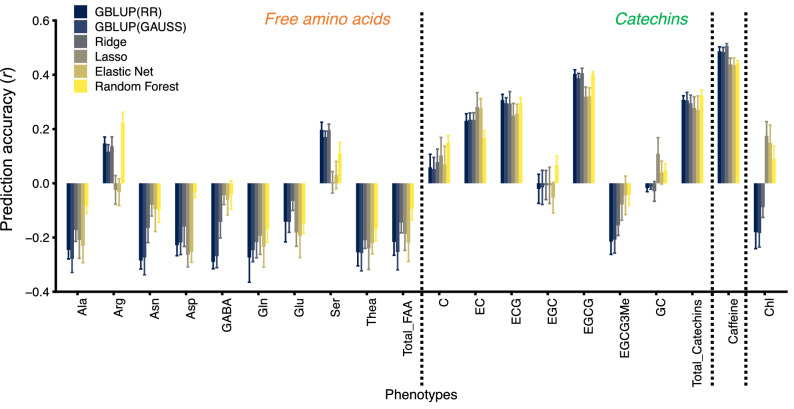
Prediction accuracy and comparison of predictive models for tea quality-related metabolites. Data and error bars are the means ± standard deviations (10 replicates of tenfold cross-validation). *RR* ridge kernel regression, *GAUSS* Gaussian kernel regression.

We evaluated the GP model accuracy for 19 tea quality-related metabolites using all 9523 genome-wide SNPs and six regression models; genomic best linear unbiased prediction (GBLUP) with linear ridge kernel regression (RR) or GBLUP with non-linear Gaussian kernel regression (GAUSS), Ridge, Lasso, Elastic Net, and Random Forest. The tenfold cross-validations (CVs) showed that the *r* values were moderate for ( −)-epicatechin (EC; *r* = 0.17–0.28), ( −)-epicatechin gallate (ECG; *r* = 0.25–0.31), EGCG (*r* = 0.32–0.41), total catechins (*r* = 0.27 − 0.32), and caffeine (*r* = 0.44 − 0.51), but low for other metabolites, such as FAAs and Chl (Fig. 3). For the five metabolites predicted with moderate accuracy by GP, the GBLUP (RR), GBLUP (GAUSS), and Ridge regression models were superior to other models (Fig. 3). These prediction values were also determined to be robust because the tenfold CVs had 10 repeats (Fig. 3).

### Estimation of the cumulative effect of GWAS-detected SNPs on the GP model

**Figure 4:**
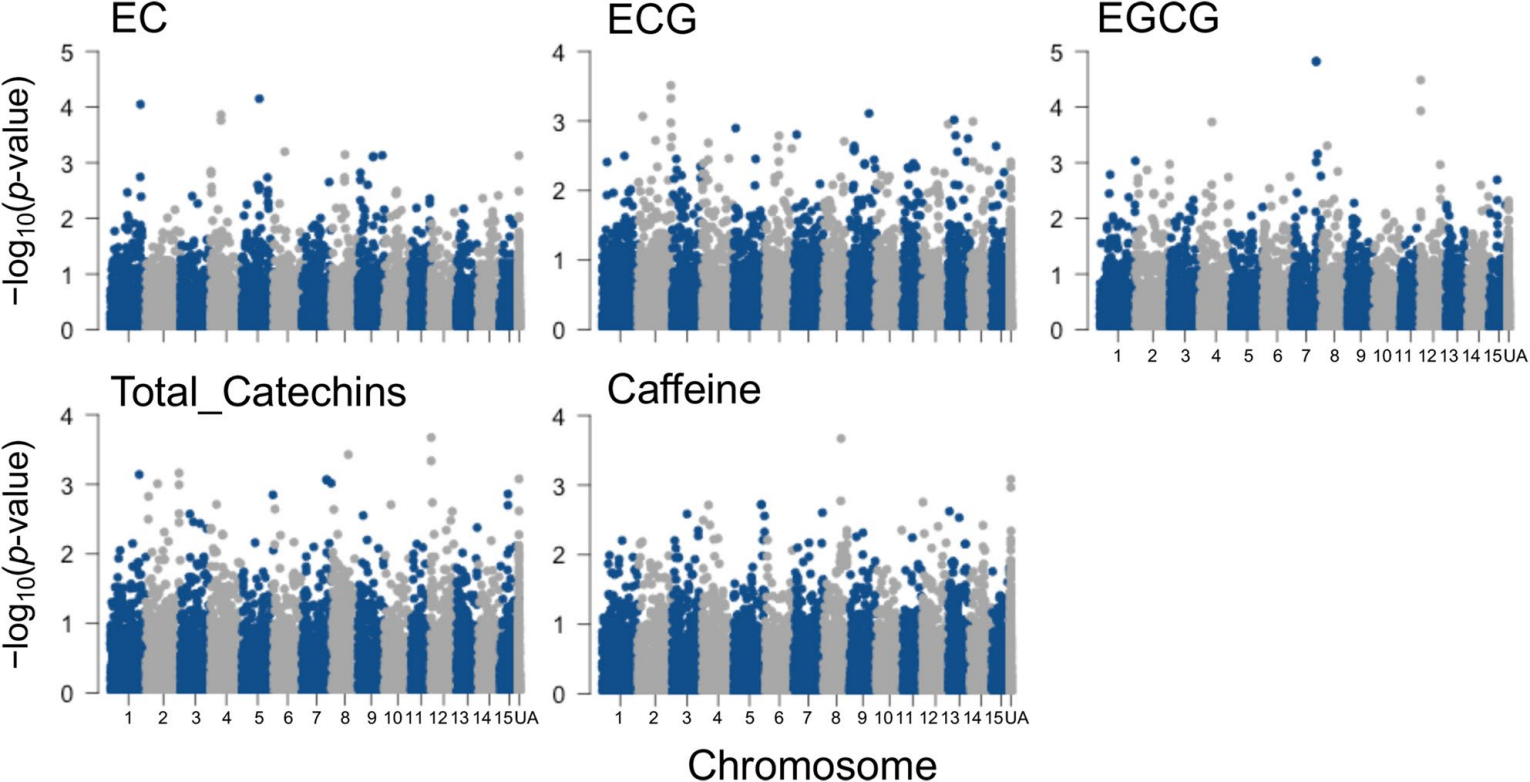
Manhattan plots from a GWAS of five phenotypes of tea quality-related metabolites. *UA* un-anchored SNPs.

**Figure 5:**
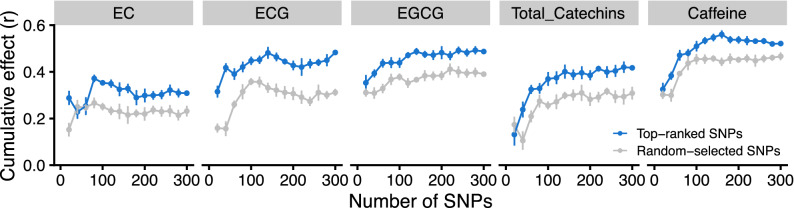
Estimation of cumulative effects of top-ranked SNPs detected by GWAS on five phenotypes of tea quality-related metabolites in the GP model. The cumulative effects of genomic prediction accuracy were evaluated using 20–300 top-ranked SNPs detected by the GWAS at 20 SNP intervals. Data and error bars are the means ± standard deviations (10 replicates of tenfold CV). 20–300 randomly selected SNPs were also used as a reference.

A GWAS was conducted for five tea quality-related metabolites that were predicted with moderate accuracy by GP (Fig. 4). A GWAS based on a mixed linear model (MLM) and general linear model (GLM) using all 9523 genome-wide SNPs identified several loci controlling each metabolite’s level (Fig. 4). Quantile–quantile (QQ) plots were used to assess the extent of accordance between the observed and expected *p*-values (Supplementary Fig. S4). The observed GLM *p*-values differed more from the expected *p*-values than those of the MLM, especially for the EGCG content phenotypes. Thus, the GWAS results based on the MLM were used for subsequent analyses. To estimate how effective GWAS-associated SNPs explained, we constructed prediction models with GBLUP (RR) that incorporated the top-ranked SNPs (i.e., SNPs with the lowest *p*-values in the GWAS) and randomly selected SNPs as a reference. The value of the *r* value curves based on top-ranked SNPs was higher than that based on randomly selected SNPs (Fig. 5). The curves of the *r* values peaked at 80–160 top-ranked SNPs per metabolite (Fig. 5), EC at 80 SNPs, ECG at 140 SNPs, EGCG at 140 SNPs, total catechins at 140 SNPs, and caffeine at 160 SNPs. Thus, relatively small numbers of top-ranked SNPs effectively explained each metabolite’s level.

We identified the potential candidate genes controlling EC (57 genes), ECG (97 genes), EGCG (64 genes), total catechins (80 genes), and caffeine (83 genes) that were located within a 10-kb region using the LD decay (Fig. 2) of the 80–160 GWAS-detected SNPs per metabolite in the 150 analysed tea accessions (Supplementary Tables S1–S5. There were 13 common candidate genes associated with the EGCG and caffeine contents (Table 1).

**Table 1:** Common candidate genes associated with EGCG and caffeine contents as assessed by genome-wide association studies (GWASs).

Associated SNPs				Candidate genes						
				GeneID	Chr	Gene position (bp)		Strand	Distance from gene (bp)	Gene annotation
Chr	Position (bp)	*P*-value for EGCG	*P*-value for caffeine			Up	Down			
12	2,549,738	3.E−05	2.E−03	CSS0013722	12	2,544,002	2,563,160	−	Region within the gene	Putative phagocytic receptor 1b
4	71,890,059	1.E−02	6.E−03	CSS0006445	4	71,892,372	71,894,683	−	2313	NA
14	87,255,362	7.E−03	2.E−02	CSS0007093	14	87,257,768	87,260,368	+	− 2406	Hypothetical protein VITISV_023007
2	12,909,634	2.E−03	7.E−03	CSS0008055	2	12,908,731	12,917,237	−	Region within the gene	Probable serine/threonine-protein kinase
7	26,653,857	1.E−02	2.E−02	CSS0012938	7	26,645,975	26,689,027	+	Region within the gene	SPA1-related 3 isoform 1
15	5,519,341	8.E−03	2.E−02	CSS0014189	15	5,518,230	5,522,057	−	Region within the gene	RING-H2 finger protein ATL3
4	71,890,059	1.E−02	6.E−03	CSS0020256	4	71,881,294	71,884,993	+	5066	Auxin response factor 17-like
1	217,617,856	8.E−03	2.E−02	CSS0023674	1	217,618,798	217,622,981	−	942	Hypothetical protein VITISV_013519
15	5,519,341	8.E−03	2.E−02	CSS0025062	15	5,502,746	5,516,691	+	2650	Ubiquitin conjugating enzyme J2
14	79,981,575	3.E−03	4.E−03	CSS0026002	14	79,978,507	79,982,432	−	Region within the gene	MATE efflux family protein 5
2	12,909,634	2.E−03	7.E−03	CSS0029046	2	12,894,561	12,904,995	−	− 4639	Proteasome subunit alpha type-2-B
4	71,890,059	1.E−02	6.E−03	CSS0030407	4	71,894,597	71,901,185	+	− 4538	Auxin response factor 17-like
2	12,909,634	2.E−03	7.E−03	CSS0050267	2	12,915,964	12,922,795	−	6330	WRKY transcription factor

## Discussion

Genomics-based approaches may be especially useful in crop breeding and reduce the required breeding time compared with conventional breeding^18^. They are effective in crops with long life cycles, such as woody plants. Tea plants have several functional metabolites that have human health benefits; therefore, establishment of new genomic breeding methods, such as GP and GWAS, is an important first step for the tea industry’s future. In the present study, we evaluated the potential of GPs and GWASs for genetic improvement of tea quality-related metabolites using genome-wide SNPs detected by RAD-seq data from 150 tea accessions. The LD pattern is a key factor for GP and GWAS because these two approaches are based on the LD between markers and polymorphisms that explain phenotypic variation^19,27^.

Of the 150 analysed tea accessions, the LD decayed within 10 kb (Fig. 2). This was greater than in a previous study, which estimated that the LD decay of tea plants was within approximately 2 kb^34^ or 5 kb^31^ using 415 or 78 accessions, respectively. This disparity may result from differences in the reference population. The present population comprised mainly composed of Japanese accessions, whereas those of previous studies^31,34^ comprised mainly Chinese accessions. In cross-pollinated species, such as tea plants, LD may decay as a result of extreme genetic drift during domestication and breeding during evolution^35–38^. The hypothesis that the progenitors of the Japanese accessions (*C. sinensis* var. *sinensis*) were introduced into Japan from China approximately 800 to 1200 years ago by Buddhist priests is supported by recent DNA marker analyses^39,40^. Therefore, these differences in LD decay values of each tea reference population may result from the Japanese tea population having a limited number of founders compared with the Chinese population. However, the mean *r*^*2*^ value (0.24) between adjacent SNPs in this population was greater than the *r*^*2*^ value (0.20) required for an accurate GP^41^. This indicated that the marker density of this population was sufficient for GP. Furthermore, the fastSTRUCTURE, hierarchical cluster analysis (HCA), and principal component analysis (PCA) results showed that these SNPs reflected sufficient genetic differentiation, similar to our previous study^28^.

We achieved moderate *r* values for EC (*r* = 0.17–0.28), ECG (*r* = 0.25–0.31), EGCG (*r* = 0.32–0.41), total catechins (*r* = 0.27–0.32), and caffeine (*r* = 0.44–0.51) from six GP models (Fig. 3). In particular, the present prediction values of the EGCG and caffeine contents, which are the most important breeding traits of tea plants, are practical and valuable. The EGCG and caffeine contents of new tea shoots must be controlled in accordance with the breeding objective. Although EGCG has strong antiallergic effects^12,13^, it is mainly responsible for the characteristic astringent and bitter taste in tea fusions^42^. Although caffeine has beneficial effects, such as reducing the risk for type 2 diabetes^14^ and increasing clear thinking and brain activity^43^, it also has harmful effects, such as the inflammation of digestive organs, insomnia, and arrhythmia^15^. Additionally, excessive intake poses risks to pregnant women^16^. GPs can accelerate breeding improvements that control taste or health benefits. However, the *r* values for the FAAs and Chl revealed negative prediction accuracies in this reference population (Fig. 3), which showed only moderate variations (Fig. 1). The *r* value for EGC also showed a negative prediction accuracy (Fig. 3). Negative prediction accuracy could be an artefact of the mathematical formulas used to calculate correlation coefficients when the expected accuracy is low^44^. One reason for the rather low FAA, Chl, and EGC *r* levels may be that their phenotypes were susceptible to environmental factors, such as light^45,46^ and temperature^47^, and tea management-related processes^48^. Because tea plants are a woody perennial crop; therefore, it is difficult to control these environmental factors. Additional reasons may be the small training population size and the traits’ genetic complexity. Thus, future challenges include resolving the effects of these factors and obtaining accurate values for breeding using a completely controlled environment, such as a large plant factory.

GWAS of five tea quality-related metabolites (EC, ECG, EGCG, total catechins, and caffeine) with high GP *r* levels using all 9,523 genome-wide SNPs identified several loci that control the level of each metabolite (Fig. 4). We estimated how effective GWAS-associated SNPs were for explaining the variation to identify the potential candidate genes. We constructed prediction models with GBLUP (RR) that incorporated the top-ranked SNPs and evaluated the curves of the *r* values that peaked in the 80–160 top-ranked SNPs per metabolite (Fig. 5). This approach might allow breeders to extract information from the training population and, simultaneously, to determine which regions of the genome are significantly associated with traits of interest, as determined by GWAS.

The potential candidate genes associated with each metabolite were detected by searching the 10-kb window (estimated LD decay region of the present 150 tea accessions; Fig. 2) based on the 80–160 top-ranked SNPs that produced the maximum cumulative prediction value (Fig. 5). The functions of most GWAS-detected candidate genes were unknown (Supplementary Tables S2–S6); therefore, their involvement in catechin and caffeine metabolism is not understood. In addition, there were 13 common candidate genes associated with the EGCG and caffeine contents (Table 1), and the EGCG and caffeine contents were positively correlated (Supplementary Fig. S5). These genes may have pleiotropic functions in each metabolite. The functions of these genes warrant further study. The present GWAS did not detect the key genes involved in the biosynthetic pathways of catechins and caffeine, such as *phenylalanine ammonia-lyase*, *chalcone isomerase*, *chalcone synthase*, *dihydroflavonol reductase*, *leucoanthocyanidin reductase*, and *anthocyanidin reductase* in catechins biosynthesis^30,49,50^ or tea caffeine synthases in caffeine biosynthesis^51,52^. This may be because of the insufficient power of the present GWAS that was conducted with multiple subpopulations to detect the subpopulation-specific alleles^53,54^, which may only segregate in some subpopulations.

The present study revealed that GP and GWAS are effective tools for genetic improvement of tea quality-related metabolites, especially the contents of several catechins and caffeine. These are pioneering results for genomics-assisted tea breeding. However, they are limited by the genetic diversity of the present Japanese tea population. We believe that this integrated GP and GWAS approach using tea accessions will be further improved by the addition of other reference populations and increased sample sizes.

## Methods

### Plant materials

Tea accessions were collected from the Tea Research Center, Shizuoka Prefectural Research Institute of Agriculture and Forestry, Kikugawa, Shizuoka, Japan. The 150 accessions comprised three subspecies: 83 Japanese var. *sinensis*, 38 exotic var. *sinensis*, and 29 Assam hybrids. Detailed additional information on the tea accessions used in this study is listed in Supplementary Table S6.

For the metabolite analysis, new shoots at the same developmental stage were harvested from 150 accessions grown in the same tea field during the first crop seasons (spring; late April to early May) of 2018 and 2019. The tea ridges were managed using conventional methods optimised for Japanese green tea cultivation. Nitrogen fertilizer was applied at 400 kg-N ha^−1^ year^−1^. New shoots were defined in this study as the upper three leaves and stems at the four-leaf developmental stage. The harvested samples were immediately placed in a cooled container (approximately 4 °C) in the field and then frozen (− 30 °C) within 1 h. After being freeze-dried, samples were ground into fine powder and stored at room temperature within a desiccator under dark conditions until the subsequent measurement of tea quality-related metabolites.

### Measurement of tea quality-related metabolites

The catechins and caffeine levels were measured. Briefly, dry ground plant tissues (25 mg) were added to 5 mL 50% acetonitrile and extracted by shaking (130 strokes per min) for 60 min at room temperature. After centrifugation (2000 × *g*, 15 min, 4 °C), the supernatants were individually passed individually through 0.45-µm polytetrafluoroethylene filters (ADVANTEC, Tokyo, Japan). The resulting solutions were stored at − 30 °C until they were analysed using high-performance liquid chromatography (HPLC). The HPLC system consisted of two LC-10ADvp pumps, a D6U-14A degasser, a CTO-20AC column oven, an SPD-M20A prominence photodiode array detector, an SCL-10Avp system controller, and an SIL-10ADvp autosampler (Shimadzu, Tokyo, Japan). The HPLC conditions used were as follows: injection volume, 5 μL; column, 75 mm × 4.6 mm × 2.6 μm SunShell C18 column (ChromaNik Technologies Inc., Osaka, Japan); column oven temperature, 40 °C; and photodiode array detector, 190 to 400 nm. Eluent A (1909:90:1 mL, ultra-pure water:acetonitrile:85% phosphoric acid) and eluent B (999:1000:1 mL, ultra-pure water:acetonitrile:85% phosphoric acid) were used as the mobile phases at a flow rate of 1.0 mL min^−1^. The elution was performed with the following gradient: initial concentration of 10% B, followed by 2.5-min hold at 10% B, 1.5-min linear gradient from 10 to 30% B, 1.0-min hold at 30% B, 2.5-min linear gradient from 30 to 80% B, 2.5-min hold at 80% B, 1.0-min gradient from 80 to 10% B, and a final concentration of 10% B for 4.0 min. The solution of this mobile phase was eluted for 15 min per sample. The seven catechins [( +)-gallocatechin, ( +)-catechin, EC, EGC, ( −)-catechin gallate, ECG, and EGCG] and caffeine were quantified, and their total value without caffeine was also expressed as total catechins.

The FAA levels were also measured. Briefly, dry ground plant tissues (10 mg) were added to 10 mg polyvinylpolypyrrolidone and 5 mL ultra-pure water and extracted by shaking (130 strokes per min) for 60 min at room temperature. After centrifugation (2000 × *g*, 15 min, 4 °C), the supernatants were independently passed through 0.45-µm cellulose acetate filters (ADVANTEC, Tokyo, Japan). The resulting solution was stored at − 30 °C until analysis by HPLC. Homoserine, as an internal standard, was added to the resulting solution, and *o-*phthalaldehyde derivatives were analysed using the HPLC system, which consisted of the following: two LC-10AT pumps, a DGU-20A5R degasser, a CTO-10Avp column oven, an RF-20A prominence fluorescence detector, an SCL-10Avp system controller, and an SIL-10AF autosampler (Shimadzu, Tokyo, Japan). The HPLC conditions used were as follows: injection volume, 5 μL; column, 75 mm × 4.6 mm × 5 μm Ascentis Express C18 column (Sigma-Aldrich, St. Louis, MO, USA); column oven temperature, 40 °C; excitation wavelength, 340 nm; and emission wavelength, 450 nm. Eluent A (5 mM citrate buffer, pH 6.0, and 5% acetonitrile) and eluent B (5 mM citrate buffer, pH 6.0, and 70% acetonitrile) were used as the mobile phases at a flow rate of 1.0 mL min^−1^. The elution was performed using the following gradient: initial concentration of 5% B, followed by 1.6-min linear gradient from 5 to 12% B, 5.0-min linear gradient from 12 to 22% B, 1.7-min linear gradient from 22 to 95% B, 2.2-min hold at 95% B, 0.5-min linear gradient from 95 to 5% B, 1.5-min gradient from 5 to 0% B, and a final concentration of 0% B for 1.0 min. The solution of this mobile phase was eluted for 15 min per sample. Nine amino acids were quantified (aspartate, asparagine, glutamate, glutamine, serine, arginine, alanine, theanine, and γ-aminobutyric acid), and their total value was also expressed as total FAAs.

In addition, Chl a and b were extracted from finely ground powder (5 mg) of freeze-dried leaf samples using *N*,*N′*-dimethylformamide (5 mL). After incubation for 24 h at 4 °C under dark conditions to allow complete decolourisation, the samples were centrifuged at 2000 × *g* for 30 min, and the absorbance of the supernatant was measured at 663.8 and 646.8 nm using a spectrophotometer (UV-1900, Shimadzu, Tokyo, Japan). The Chl *a* and *b* contents were calculated using the equation of Porra et al. (1989)^55^, and their total value was expressed as the Chl content.

### SNP genotyping data

In a previous study, we obtained the sequencing reads for SNP genotyping using RAD-seq for a tea population, which included the 150 accessions tested in this study^28^. Reads were pre-processed using Trimmomatic ver. 0.33 with the following parameters: ‘ILLUMINACLIP TruSeq3-PE-2.fa’, 2:30:10; ‘LEADING’, 19; ‘TRAILING’,19; ‘SLIDINGWINDOW’,30:20; ‘AVGQUAL’, 20; and ‘MINLEN’, 51. After pre-processing, the remaining reads were mapped to the tea reference chromosome-scale genome, which was downloaded from the Tea Plant Information Archive^31,56^ using Bowtie2 ver. 2.3.5.1, and then, the SNPs were called using Stacks ver. 2.5^57^. For subsequent analyses, SNP genotypes were converted to 1 (AA homozygotes), − 1 (BB homozygotes), or 0 (AB heterozygotes). The raw SNP data were filtered using VCFtools ver.0.1.16 with the following thresholds: SNP call rate within a locus ≥ 0.7 and MAF ≥ 0.05. The filtered SNP data were imputed using the R package missForest^58^ ver. 1.4 and used for subsequent genetic analyses. The RAD-seq data have been deposited in the DDBJ Sequence Read Archive (Accession number: DRA008166).

The LD values between pairs of SNPs in the same chromosome were determined from the squared correlation coefficients (*r*^2^) values within the 50-kb window using VCFtools ver. 0.1.16. Pairwise LDs were plotted against physical distances. The LD decay pattern was estimated by fitting a trend line based on a nonlinear LOESS regression of *r*^2^ on physical distance using the R package ggplot2 ver. 3.3.2. Physical distances between adjacent markers ranged from 1.00 to 49.97 kb (mean, 10.75 kb). The genetic structure and admixture of all 150 accessions can be found in our previous study^28^.

To clarify the genetic structure, we used the Bayesian clustering algorithm, HCA, and PCA. The Bayesian clustering analysis was performed using fastSTRUCTURE ver. 1.0^59^. The components of each subgroup, that is ancestral components, determined by this fastSTRUCTURE analysis were compared with the genetic structure information from our previous study (group1, 2, and 3)^28^. HCA was based on Ward’s method^60^ using Euclidean distance and was conducted using the R function “hclust”. PCA was performed using the R function prcomp. The principal component scores were plotted using R package ggplot2 ver. 3.3.2.

### GP models

To evaluate the GP accuracy, we used six regression methods. In phenotype value, the mean values obtained from 2018 and 2019 were used as values in the subsequent GP modelling and GWAS. The GBLUP with RR and GAUSS were performed using the “kinship.BLUP” function of the R package rrBLUP ver. 4.6.1^61^. Restricted maximum likelihood was used to estimate variance components. Ridge (alpha = 0), Lasso (alpha = 1), and Elastic Net (alpha = 0.5) were performed as linear regression methods using the R package glmnet ver. 4.0^62^. Random Forest, a non-linear decision tree-based ensemble learning method, was performed using the R package randomForest ver. 4.6-14^63^. Number of trees (ntree) was set to 1000, and default values were used for the other parameters.

The *r* values of the models were estimated using 10 replicates of tenfold CVs. Thus, *r* was defined as Pearson’s correlation coefficient between observed and predicted values. Using tenfold CVs, the entire population was divided into 10 equal groups. One group was predicted by regression models based on the other nine groups (reference populations). Correlations were calculated until all individuals in all groups received phenotypic predictions. Then, a single correlation was calculated between all observed and predicted phenotypes within all groups.

### Cumulative effect estimation and candidate genes identification using GWAS-detected SNPs

The GWAS was performed using an MLM implemented using the “GWAS” function of the R package rrBLUP ver. 4.6.1^61^. In total, 9,523 SNPs were used for the GWAS after selecting SNPs without missing rates ≥ 0.3 and MAFs < 0.05. The principal components and a kinship matrix were included in the GWAS calculation based on the MLM. A PCA was conducted using the R function “prcomp” to estimate the population structure. The first six principal components from the plot that explained the total variation among SNPs were selected. The kinship matrix was computed using the “A.mat” function of the R package rrBLUP ver. 4.6. To illustrate the localisation of associated SNPs by GWAS, we created Manhattan plots from SNPs anchored to 15 chromosomes and 1,318 unanchored contigs. The Manhattan plots were illustrated using the “manhattan” in the R package qqman ver.0.1.4^64^.

To estimate how effective the SNPs were in explaining the variation and to validate the potential of the GWAS-associated SNPs in the GP model, a GP analysis by GBLUP (RR) was performed using the SNPs with the lowest *p*-values (top-ranked) in the GWAS, as described by Nakano et al. (2020) with slight modifications^65^. The cumulative effects of the linked loci were estimated using 20–300 top-ranked SNPs (at 20 SNPs intervals). Randomly selected SNPs throughout the genome were used as a reference. The potential candidate genes that were located within a 10-kb region using the LD decay (Fig. 2) of the 80–160 GWAS-detected SNPs per metabolite (Fig. 5) were screened based on the gene annotations in the tea reference chromosome-scale genome, which was downloaded from the Tea Plant Information Archive^31,56^.

## Supplementary information

Supplementary Tables.

Supplementary Figures.
