# Supplementary material for: Genomic predictions and genome-wide association studies based on RAD-seq of quality-related metabolites for the genomics-assisted breeding of tea plants
Source: Sci Rep. 2020 Oct 15;10:17480. doi: 10.1038/s41598-020-74623-7 (PMC7562905; doi:10.1038/s41598-020-74623-7)
Supplement: Supplementary file 1 — Supplementary Tables. [file 41598_2020_74623_MOESM1_ESM.pdf]

Supplementary Information

Genomic predictions and genome-wide association studies based on RAD-seq of quality-related metabolites for the genomics-assisted breeding of tea plants

Hiroto Yamashita, Tomoki Uchida, Yasuno Tanaka, Hideyuki Katai, Atsushi J. Nagano, Akio Morita, Takashi Ikka

*Corresponding author
Email: ikka.takashi@shizuoka.ac.jp (TI)

<u>This PDF file includes:</u>

Supplementary Figure S1. Correlations of tea quality-related metabolites in 150 accessions between 2018 and 2019. NS; Not significant ($P > 0.01$)

Supplementary Figure S2. Distribution of genome-wide SNPs in the tea chromosome-scale genome. UA; Un-anchored SNPs.

Supplementary Figure S3. Genetic structure analyses using 9,523 SNPs among 150 tea accessions. Estimated genetic structure for K = 2 and K = 3 by fastSTRUCTURE (A). Plot of first and second principal components computed by PCA (B). Dendrogram of Ward's hierarchical clustering based on Euclidean distance (C).

Supplementary Figure S4. QQ-plots from a GWAS based on the MLM (A) and GLM (B) of five phenotypes of tea quality-related metabolites.

Supplementary Figure S5. Correlation matrix between tea quality-related metabolites.

<u>Supplementary Data file includes:</u>

Supplementary Table S1. List of candidate genes linked to the top 80 SNPs in a GWAS of EC content.

Supplementary Table S2. List of candidate genes linked to the top 140 SNPs in a GWAS of ECG content.

Supplementary Table S3. List of candidate genes linked to the top 140 SNPs in a GWAS of EGCG content.

Supplementary Table S4. List of candidate genes linked to the top 140 SNPs in a GWAS of total catechin content.

Supplementary Table S5. List of candidate genes linked to the top 160 SNPs in a GWAS of caffeine content.

Supplementary Table S6. Tea accessions used in this study.

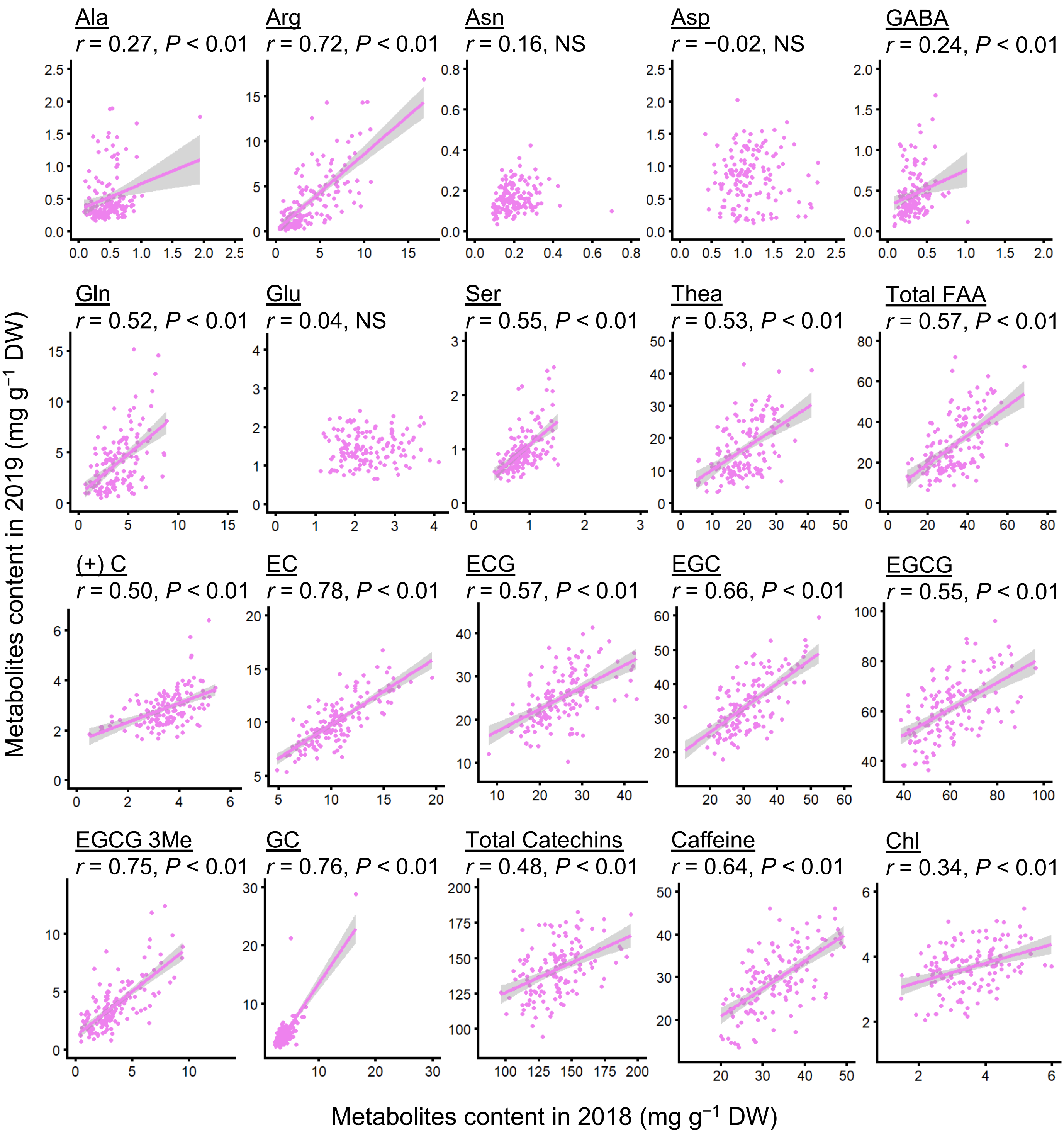

Supplementary Figure S1. Correlations of tea quality-related metabolites in 150 accessions between 2018 and 2019.
NS; Not significant ($P > 0.01$)

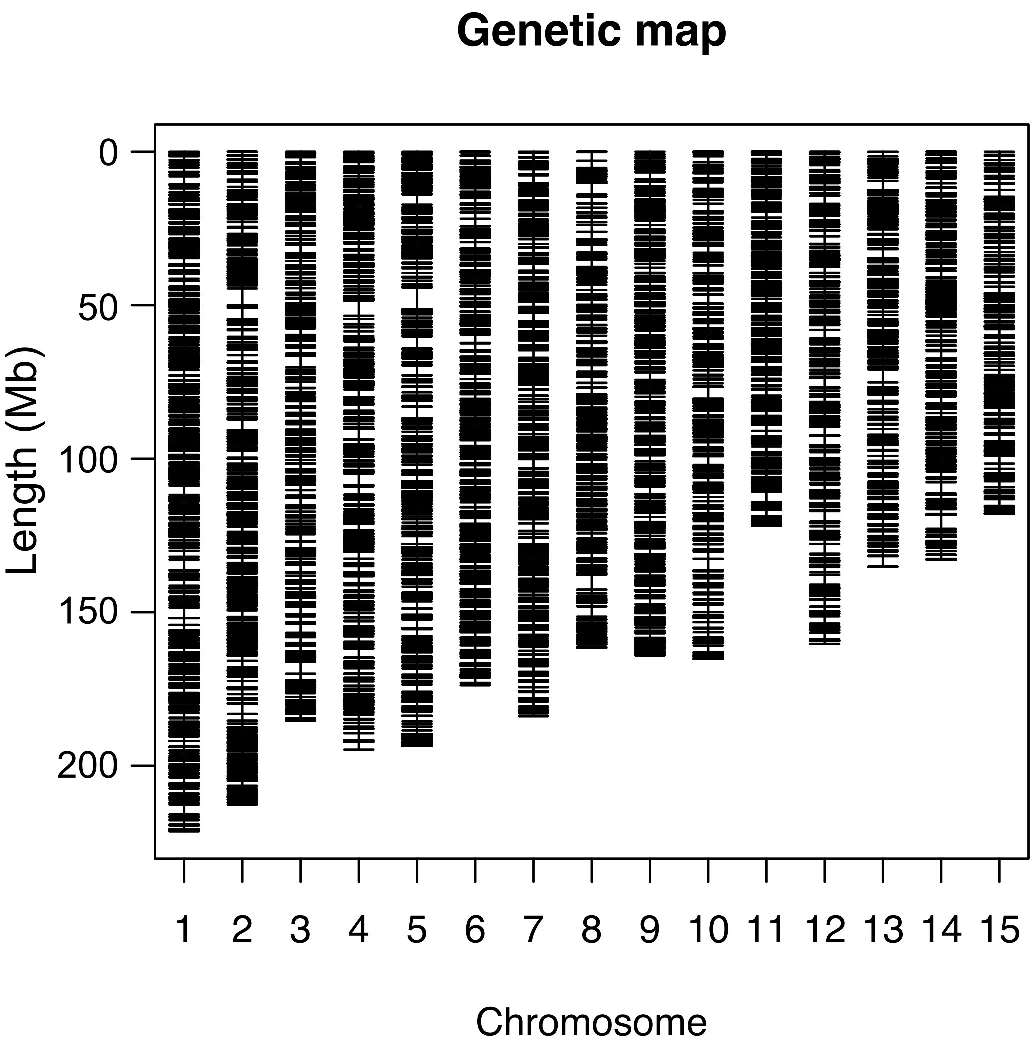

Chr	Number of SNPs
1	911
2	781
3	571
4	606
5	626
6	612
7	609
8	541
9	639
10	480
11	485
12	521
13	493
14	446
15	380
UA	822
Total	9,523

Supplementary Figure S2. Distribution of genome-wide SNPs in tea chromosome-scale genome. UA; Un-anchored SNPs.

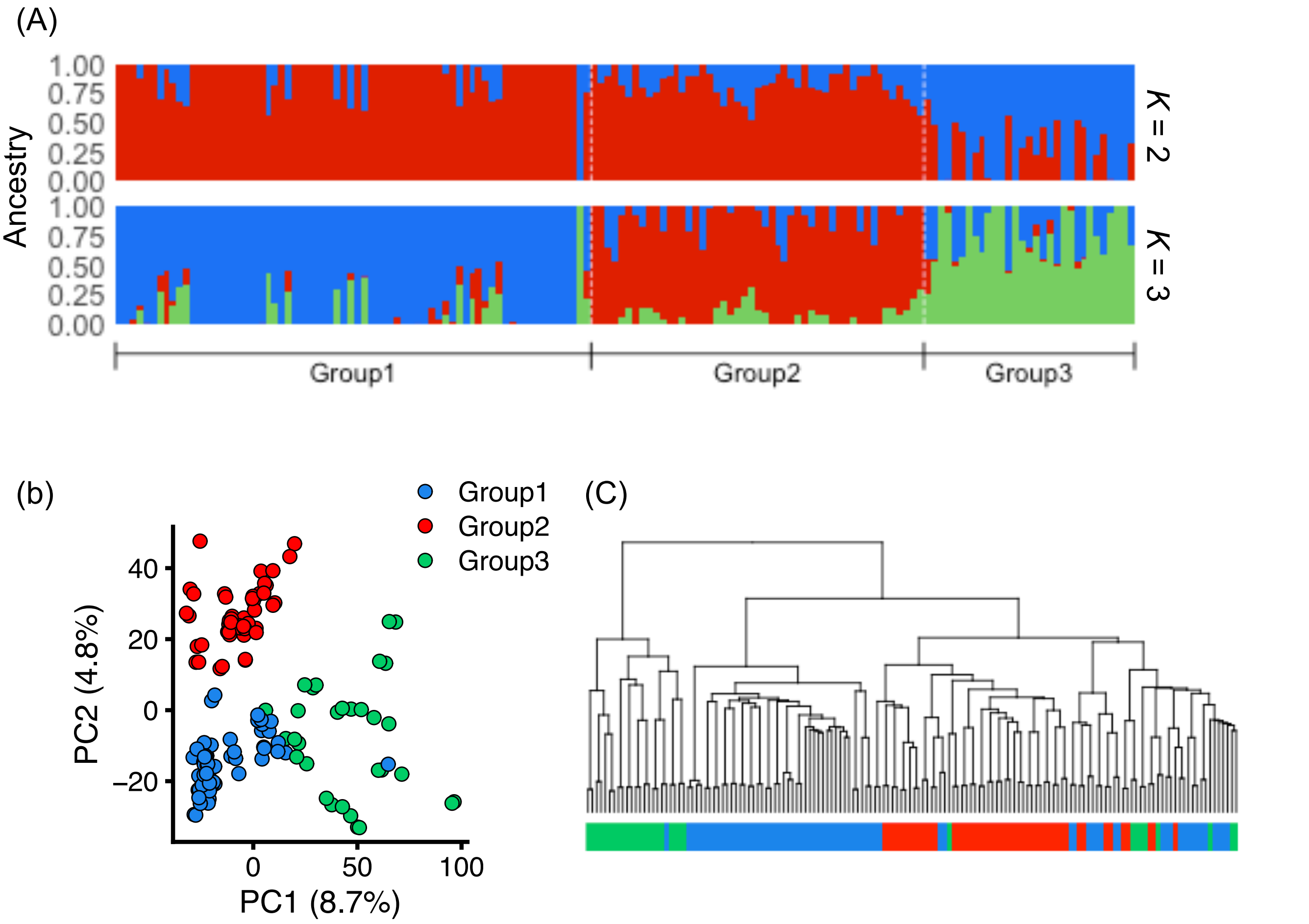

Supplementary Figure S3. Genetic structure analyses using 9,523 SNPs among 150 tea accessions. Estimated genetic structure for *K* = 2 and *K* =3 by fastSTRUCTURE (A). Plot of first and second principal components computed by PCA (B). Dendrogram of Ward's hierarchical clustering based on Euclidean distance (C).

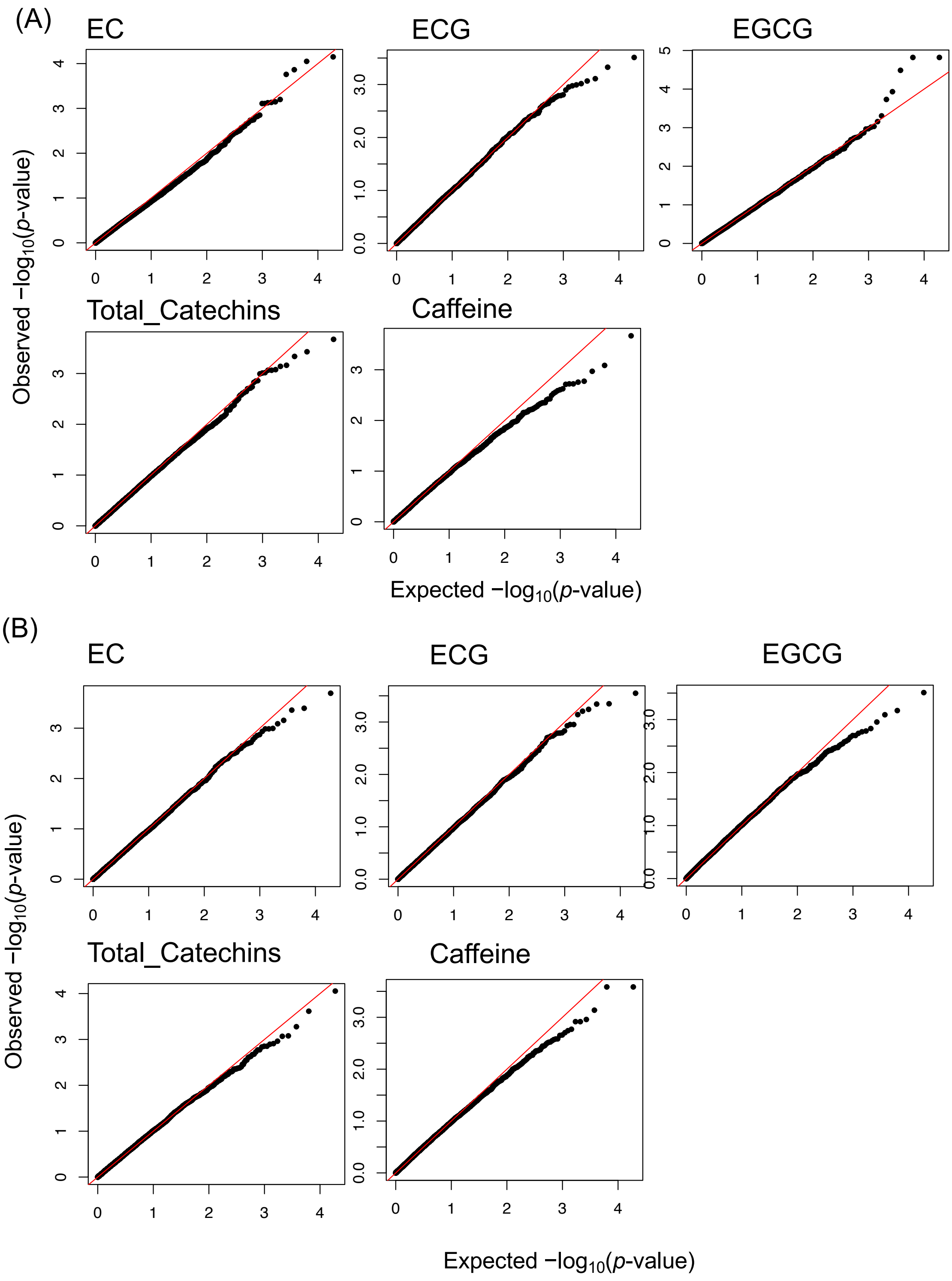

Supplementary Figure S4. QQ-plots from a GWAS based on the MLM (A) and GLM (B) of five phenotypes of tea quality-related metabolites.

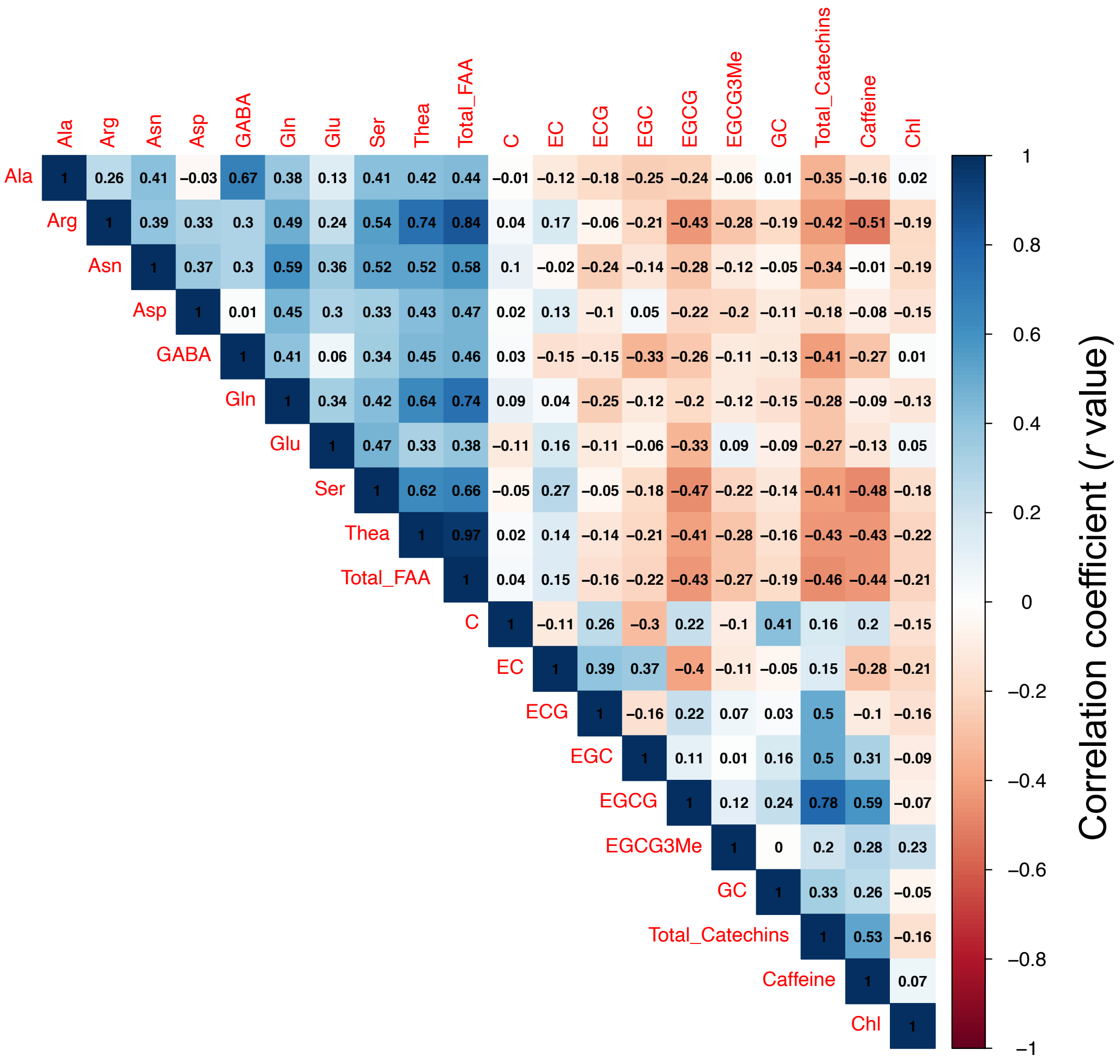

Supplementary Figure S5. Correlation matrix between tea quality-related metabolites.
